# Antibacterial Activity of Novel Agent N-2-Hydroxypropyl Trimethyl Ammonium Chloride Chitosan against *Streptococcus mutans*

**DOI:** 10.3390/molecules29174126

**Published:** 2024-08-30

**Authors:** Yuan Gao, Xiaochen Gong, Qicheng Ruan, Chunjing Zhang, Kai Zhao

**Affiliations:** 1 Zhejiang Provincial Key Laboratory of Plant Evolutionary Ecology and Conservation, Taizhou Key Laboratory of Biomedicine and Advanced Dosage Forms, School of Life Sciences, Taizhou University, Taizhou 318000, China; gaoyuan@tzc.edu.cn (Y.G.); m17865925682@163.com (X.G.); ruanqicheng@163.com (Q.R.); 2 School of Medical Technology, Qiqihar Medical University, Qiqihar 161006, China

**Keywords:** dental caries, antibacterial, N-2-HACC, *Streptococcus mutans*

## Abstract

Dental caries (DC) is one of the most common oral diseases and is mainly caused by *Streptococcus mutans* (*S. mutans*). The use of antibiotics against *S. mutans* usually has side effects, including developing resistance. N-2-Hydroxypropyl trimethyl ammonium chloride chitosan (N-2-HACC), a natural product, has great potential utility in antibacterial agents owing to its low toxicity and good biocompatibility. Thus, the purpose of the present study was to explore the antimicrobial activity of N-2-HACC against *S. mutans* through the permeability of the cell wall, integrity of cell membrane, protein and nucleic acid synthesis, respiratory metabolism, and biofilm formation. Our results confirmed that the MIC of N-2-HACC against *S. mutans* was 0.625 mg/mL with a 90.01 ± 1.54% inhibition rate. SEM observed the formation of cavities on the surface of *S. mutans* after 12 h N-2-HACC treatment. The level of alkaline phosphatase (AKP) activity was higher in the N-2-HACC treatment group than in the control group, indicating that N-2-HACC can improve the permeability of the cell wall. Also, N-2-HACC treatment can destroy the cell membrane of *S. mutans* by increasing conductivity and absorbance at 260 nm, decreasing cell metabolic activity, and enhancing the fluorescence at 488 nm. Respiratory metabolism revealed that the activities of the Na^+^-K^+^-ATP enzyme, pyruvate kinase (PK), succinate dehydrogenase (SDH), and malate dehydrogenase (MDH) were decreased after N-2-HACC treatment, revealing that N-2-HACC can inhibit glycolysis and the tricarboxylic acid cycle (TCA cycle) of *S. mutans*. Moreover, N-2-HACC can also decrease the contents of the nucleic acid and solution protein of *S. mutans*, interfere with biofilm formation, and decrease the mRNA expression level of biofilm formation-related genes. Therefore, these results verify that N-2-HACC has strong antibacterial activity against *S. mutans*, acting via cell membrane integrity damage, increasing the permeability of cell walls, interfering with bacterial protein and nucleic acid synthesis, perturbing glycolysis and the TCA cycle, and inhibiting biofilm formation. It is suggested that N-2-HACC may represent a new potential synthetically modified antibacterial material against *S. mutans*.

## 1. Introduction

Nearly half of the global population suffers from oral diseases, which are among the largest public health problems worldwide. Oral diseases may lead to tooth loss, persistent pain, and impaired masticatory function; reports even show that oral diseases and chronic systemic diseases influence each other [1,2]. Dental caries (DC) is a typical oral disease with high incidence and prevalence that affects the oral health of both children and adults. DC can be caused by bacteria, saliva, and food in dental plaque; it is characterized by the resultant lesions arising from pH fluctuations [2,3]. Bacteria, including *Streptococcus mutans* (*S. mutans*) and *Streptococcus sobrinus* (*S. sobrinus*), can cause DC [2]. However, *S. mutans* is a major etiological agent of human DC; it can easily attach to the tooth surface, rapidly metabolize a wide variety of carbohydrates to produce acid, and survive in low-pH environments, leading to the dissolution of the hydroxyapatite of tooth enamel and damaging the tooth surface, even resulting in tooth loss [4,5,6].

Synthetic drugs are the main way to inhibit *S. mutans* biofilm formation. However, concerns about the chemical toxicity and potentially different side effects of medicines used against DC have led to a growing call for the use of ingredients derived from natural resources. As an amino-abundant natural polysaccharide, chitosan is a deacetylated product of chitin [7,8]. Chitosan has been shown to have numerous advantages and properties, including low toxicity, biocompatibility, biodegradation, and anti-inflammatory and antioxidant effects, in medicine and biomedical applications [9,10]. It can act against bacteria by destroying the integrity of the biofilm and promoting the decomposition of the biofilm [11]. However, the pH-dependent water solubility and poor mechanical strength of chitosan limit its application. The chemical modification of chitosan can not only help retain its original properties but also enhance its solubility [12].

Quaternization is a major method that can be used for enhancing the solubility of chitosan [12]. Quaternized chitosan is a cationic antibacterial agent and is widely applied as a promising polysaccharide for its broad-spectrum antibacterial properties [9,13,14]. N-2-Hydroxypropyl trimethyl ammonium chloride chitosan (N-2-HACC) is a kind of positively charged quaternized chitosan that is more water-soluble than chitosan, enables convenient synthesis, and entails lower costs than chitosan [7]. Importantly, the antibacterial activity of N-2-HACC is greatly improved because of the introduction of quaternary phosphonium salt. A previous study demonstrated that N-2-HACC has very low toxicity and high safety levels [15]. Chitosan quaternary ammonium salt has a good inhibitory effect on bacteria and fungi, including *Staphylococcus aureus*, *Escherichia coli*, *Botrytis cinerea*, *S. mutans*, and *Fusarium oxysporum f. sp. Cubense* [16,17]. The modified polysaccharides can also remove Microcystis aeruginosa cells from drinking water [18]. Moreover, N-2-HACC has great application prospects in drug or vaccine delivery systems and as a vaccine adjuvant [7,19,20,21]. To our knowledge, there are few reports about the antibacterial activity of N-2-HACC as an antibacterial polysaccharide active against *S. mutans*.

The purpose of this study was to synthesize a safe and water-soluble N-2-HACC and explore its antibacterial activity against *S. mutans* as it relates to the permeability of the cell wall, the integrity of the cell membrane, protein and nucleic acid synthesis, respiratory metabolism, and biofilm formation. These provide valuable insights into the further development and application of the antibacterial activity of natural products.

## 2. Results

### 2.1. Characteristics of Antibacterial of N-2-HACC

**Figure 1:**
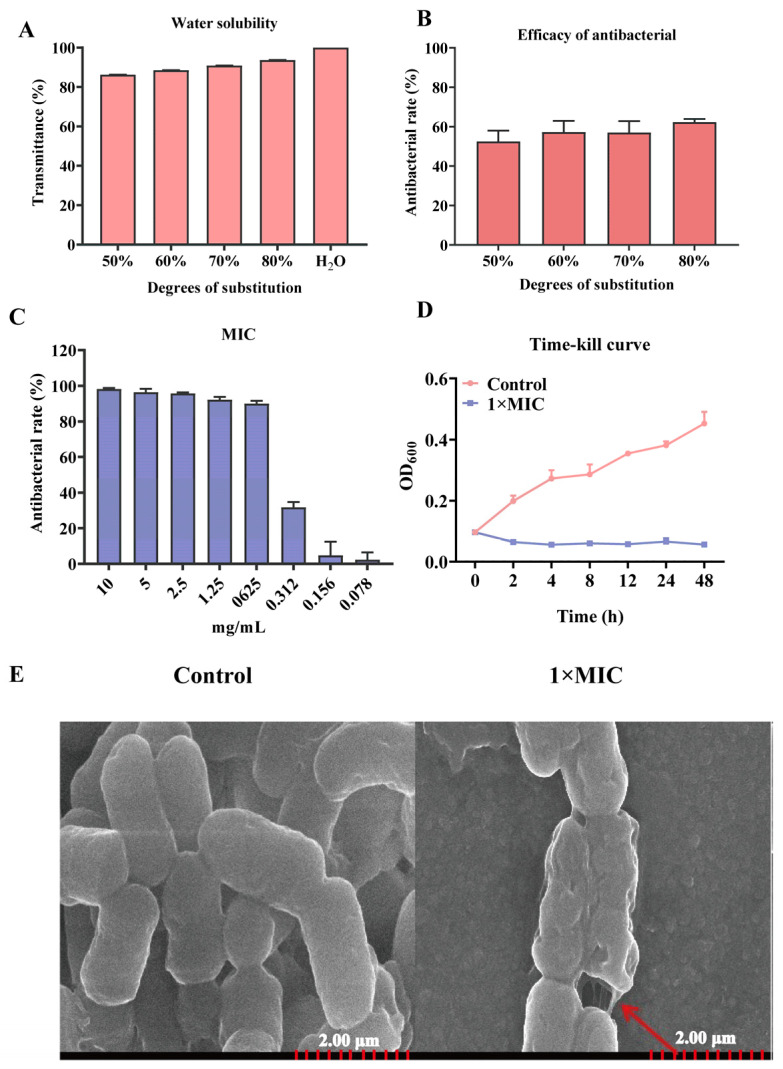
The antibacterial effects of N-2-HACC. (**A**) Water solubility of N-2-HACC; (**B**) antibacterial effects of N-2-HACC; (**C**) MIC of N-2-HACC; (**D**) time–kill curve of N-2-HACC; (**E**) SEM observation of *S. mutans* morphology. Bar = 2.00 μm.

In the present study, four kinds of N-2-HACC were synthesized with 50%, 60%, 70%, and 80% degrees of substitution. The results of the water solubility showed that the 80% substitution of N-2-HACC had more than 80% transmittance, indicating that it was more soluble in water than the other degrees of substitution of N-2-HACC (Figure 1A). The antibacterial rate of N-2-HACC was 62.40 ± 1.55% for 80% substitution, which was higher than that for N-2-HACC with 50%, 60%, and 70% degrees of substitution (Figure 1B). Thus, an 80% substitution of N-2-HACC was used in the present study.

The MIC of N-2-HACC against *S. mutans* was 0.625 mg/mL, and this could inhibit 90.01 ± 1.54% growth of bacteria (Figure 1C). Compared with the control group, the time–kill curve showed that *S. mutans* could be effectively inhibited by N-2-HACC (Figure 1D).

SEM was employed to observe the cell membrane morphology of *S. mutans*, and the results showed that the structure of *S. mutans* underwent significant changes, including the formation of cavities after 12 h of treatment with N-2-HACC at 1× MIC (Figure 1E).

### 2.2. Effect of N-2-HACC on the Bacterial Cell Membrane and Cell Wall

**Figure 2:**
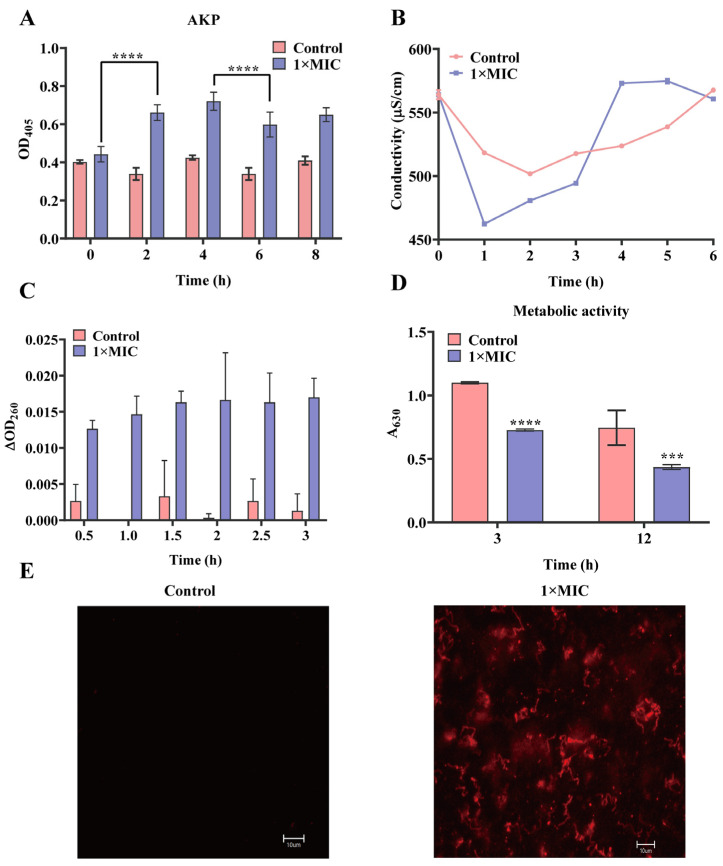
The effect of N-2-HACC on bacterial cell membrane and cell wall. (**A**) Change in cell wall after treatment with N-2-HACC. (**B**) Measurement of conductivity. (**C**) Nucleic acid leakage of *S. mutans*. (**D**) Metabolic activity of *S. mutans*. (**E**) Fluorescent microscope observation of *S. mutans*. Bar = 10.00 μm. **** *p* < 0.0001, *** *p* < 0.001.

The permeability of the cell wall was evaluated by the content of AKP in *S. mutans* after being treated with N-2-HACC. The results showed that the activity of AKP at 2 h was significantly higher than that at 0 h (*p* < 0.0001), proving that the permeability of the bacterial cell wall was increased after 2 h of treatment with N-2-HACC (Figure 2A).

The conductivity of the suspension of *S. mutans* was increased after 3 h of treatment with N-2-HACC compared with the control group (Figure 2B). Absorbance at 260 nm for the 1× MIC N-2-HACC group was higher than for the control group, indicating the nucleic acid of *S. mutans* was leaked after N-2-HACC treatment (Figure 2C).

Cell metabolic activity assessments showed that the A_630_ of 1× MIC N-2-HACC group was 0.727 ± 0.008 at 3 h, which was lower than that of the control group (*p* < 0.0001) (Figure 2D). Fluorescence at 488 nm of PI stain was also observed in the treatment groups of 1× MIC N-2-HACC, rather than the control group (Figure 2E). These results demonstrate that N-2-HACC can destroy the cell membrane of *S. mutans*.

### 2.3. Effect of N-2-HACC on Bacterial Respiratory Metabolism

**Figure 3:**
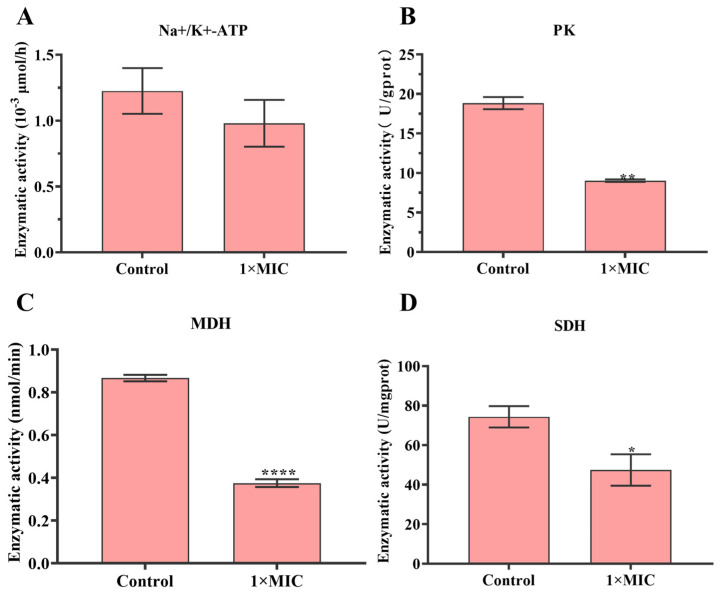
The effect of N-2-HACC on *S. mutans* respiratory metabolism. (**A**) Na^+^-K^+^-ATPase. (**B**) Pyruvate kinase, PK. (**C**) Malate dehydrogenase, MDH. (**D**) Succinate dehydrogenase, SDH. * *p* < 0.05, ** *p* < 0.01, **** *p* < 0.0001.

Glycolysis and tricarboxylic acid cycle (TCA cycle) are important systems in a living organism. The activities of Na^+^-K^+^-ATP, PK, MDH, and SDH were detected in *S. mutans*. The results showed that the activity of Na^+^-K^+^-ATP, PK, MDH, and SDH in the N-2-HACC treatment group was lower than that in the control group after 12 h of N-2-HACC incubation (Figure 3).

### 2.4. Effect of N-2-HACC on Bacterial DNA and Solution Protein

**Figure 4:**
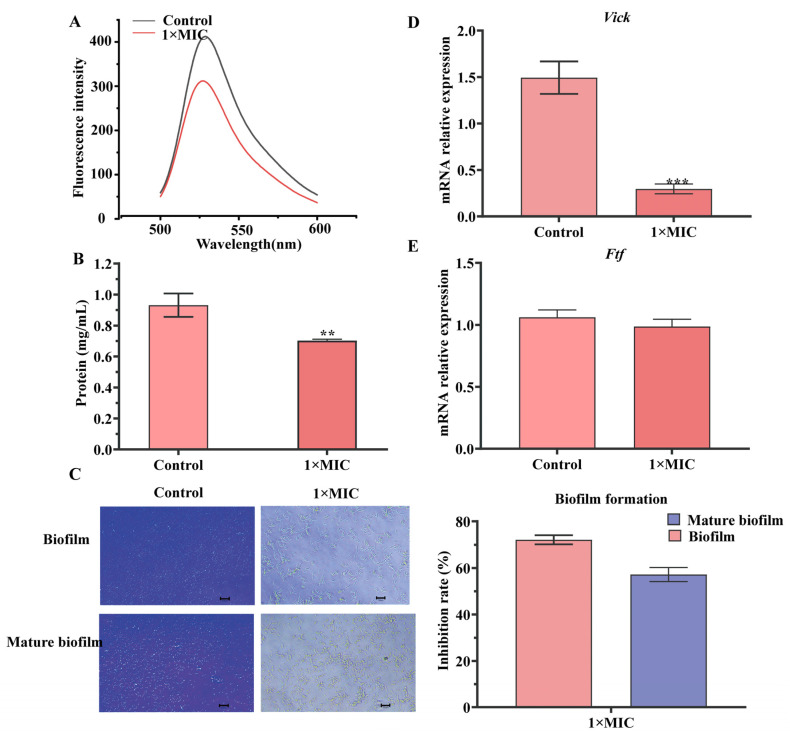
The effect of N-2-HACC on *S. mutans*. (**A**) Fluorescence spectrum of EB-DNA-N-2-HACC. (**B**) Change in soluble protein. (**C**) Formation of biofilm and inhibition rate of biofilm. (**D**,**E**) Relative *vick*, *ftf* mRNA expression. ** *p* < 0.01, *** *p* < 0.001.

DNA–ethidium bromide (EB) complexes treated with N-2-HACC showed a more obvious fluorescence quenching compared with the control group (Figure 4A). The BCA assay results showed that N-2-HACC can more significantly decrease the contents of the solution protein of *S. mutans* compared with the control group (Figure 4B).

### 2.5. Effect of N-2-HACC on Biofilm Formation

The effects of N-2-HACC at 1× MIC on the biofilm formation of *S. mutans* were determined by crystalline violet staining. As shown in Figure 4E, the inhibitory rates of 1× MIC N-2-HACC were 72.18 ± 1.96% for biofilm and 57.18 ± 2.99% for mature biofilm, respectively (Figure 4C). The results show that compared with the control group, N-2-HACC can effectively interfere with the formation of biofilm.

*Vick* and *ftf* are two key genes that act during biofilm formation. The relative expressions of *vick* and *ftf* of *S. mutans* in the N-2-HACC-treated group were decreased compared with those of the control group, indicating that N-2-HACC can inhibit the virulence of *S. mutans* (Figure 4D,E).

## 3. Discussion

*S. mutans* is the most important pathogen in the development of DC [2,22]. Antibiotics are usually used against it to minimize dental caries in clinic [23,24]. However, this medicine can affect cellular functions, causing the bacteria to develop resistance [3,25]. Effective prevention, rather than treatment, has been identified as the key element of DC management. N-2-HACC, a chitosan derivative, has potential utility in natural bioactive substances as an effective antibacterial agent. However, its antibacterial activity has not been clarified yet. Thus, in the present study, we explored the antibacterial activity of N-2-HACC with reference to the permeability of the cell wall, the integrity of the cell membrane, protein and nucleic acid synthesis, respiratory metabolism, and biofilm formation.

Usually, the antimicrobial mechanisms of antibiotics are divided into five stages, including the inhibition of cell wall synthesis, the depolarization of the cell membrane, the inhibition of protein synthesis, the inhibition of nucleic acid synthesis, and the inhibition of metabolic pathways [26]. N-2-HACC, a kind of bioactive polysaccharide, has a positive charge and can be adsorbed into the negative *S. mutans* to depolarize the cell membrane and destroy its inherent integrity [8,27]. MIC is the most basic and key microbiological parameter used to verify antibacterial potential [28]. Previous studies have shown that many natural products have potential antimicrobial activity against *S. mutans*; for example, the MIC of *Crataegi fructus* extract against *S. mutans* was 30 mg/mL, and *Cyperus articulatus* (priprioca) extract has an antimicrobial activity against *S. mutans* of 0.29 mg/mL [2,29]. Moreover, it has been reported that modified chitosan (benzoyl chitosan) has a higher antibacterial rate than chitosan [11]. In the present study, the MIC of N-2-HACC against *S. mutans* was 0.625 mg/mL, and this can inhibit its growth for 48 h, indicating that N-2-HACC has strong antimicrobial activity against *S. mutans* and represents a potential candidate for an antimicrobial agent.

The cell wall is essential to the survival and colonization of bacteria, as it can form a barrier for antimicrobial agents [30]. AKP activity is used to evaluate the integrity of the cell wall [31]. Cell membranes are a selective osmotic barrier that can shield cells from harmful substances while permitting the passage of essential nutrients required for survival [31]. The leakage of nucleic acids or proteins from the cell is often a critical sign of cell membrane integrity [31,32]. SEM revealed that the morphological structure of *S. mutans* changed, with wrinkles and cavities developing on the bacterial surface, after treatment with N-2-HACC. These results are consistent with those of a previous study, which used chitosan–gentamicin against *Vibrio parahaemolyticus* [33]. Moreover, the variations in AKP activity, metabolic activity, conductivity, PI strains, nucleic acid, and protein of *S. mutans* after N-2-HACC treatment in the present study all indicate that N-2-HACC can destroy the structure of *S. mutans* and increase the permeability of the cell wall and the integrity of the cell membrane. These results are similar to those of a previous study, in which chitosan derivatives were shown to significantly increase the protein content in *E. coli* and *S. aureus* suspensions [12]. Moreover, chitosan can destroy the integrity of the cell membranes of *Burkholderia pseudomallei* and *Lactobacillus plantarum*, causing the leakage of nucleic acid and protein [34,35].

Adenosine triphosphate (ATP) is considered to be an important compound in many activities; it can provide energy for cells, participate in the overall energy balance, and maintain intracellular homeostasis [36]. ATPase is a necessary enzyme that can catalyze ATP to ADP, and thus perform physiological functions. The decline of ATPase activity can prevent cell growth and even lead to cell death [37]. The decrease in the level of ATPase in *S. mutans* treated with N-2-HACC indicated that the activity of ATPase was destroyed by N-2-HACC. These results are similar to those for *Prunella vulgaris L.* against methicillin-resistant *S. aureus* and linalool against *Pseudomonas fluorescens*, which can both inhibit the metabolic respiration of bacteria by reducing the activity of ATPase [31,36]. TCA cycle functions as the main pathway of respiratory energy production and the central hub of metabolism in living organisms [31]. The weakening of the TCA cycle may lead to a decrease in cellular respiration and energy production and even cause organism death [38]. PK, SDH, and MDH are the key enzymes related to the TCA cycle, and they play a decisive role in glycolysis [36]. The results related to bacterial respiratory metabolism indicated that, in comparison with the control group, the levels of PK, SDH, and MDH decreased after being treated with N-2-HACC, demonstrating that N-2-HACC can inhibit the glycolysis and TCA cycle of *S. mutans*.

Biofilm is an extracellular aggregate that can protect internal bacteria from changes in the external environment [11]. Usually, *S. mutans* is recognized as a biofilm promoter [29,30]. It can survive and grow in the acidic environments of biofilm, leading to severe DC. In the present research, the inhibitory rate of biofilm formation and the level of mRNA of biofilm-related genes in the N-2-HACC-treated group were decreased compared with those of the control group, indicating that H-2-HACC has an anti-biofilm effect. The results also stand for benzoyl chitosan, which can inhibit biofilm removal in *S. aureus* by 41.5–69.9% [11].

## 4. Materials and Methods

### 4.1. Synthesis of N-2-HACC with Different Degrees of Substitution

N-2-HACC (50%, 60%, 70%, and 80% degrees of substitution) were synthesized using chitosan (with an average molecular weight of 71.3 kDa and a deacetylation degree of 80%, Sinopharm Chemical Reagent Co., Ltd., Shanghai, China) and 2,3-epoxypropyltrimethylammonium chloride (EPTAC, Shanghai Yi’en Chemical Company, Shanghai, China), as previously described [15]. Briefly, a mixture of EPTAC (5.45–11.5 g used for different degrees of substitution), chitosan, and isopropanol was poured into a three-neck flask. The mixture was heated to 80 °C under constant stirring until dissolved. After continuing the reaction for 9 h, 150 mL of ethanol was added at room temperature. The products were dialyzed, concentrated, and dried; a 0.05 mol/L silver nitrate (AgNO_3_) solution was used for titration to calculate the degree of substitution (1). The 1H NMR spectrum of the resulting polymers was similar to the spectrum of these polymers obtained using a previously published method [15].

(1) $$\mathrm{Degrees}\;\mathrm{of}\;\mathrm{substitution}=V\times C\times 10^{-3}÷V\times C\times 10^{-3}-\left(W-V\times C\times 10^{-3}\times M_{\text{N-2-HACC}}/M_{\mathrm{CS}}\right)$$

Here, C is the molarity of the AgNO_3_ solution (mol/L); V is the volume of the AgNO_3_ solution (mL); W is the quality of N-2-HACC (g); M_N-2-HACC_ is the molecular weight of the chitosan monomer substituted by quaternary ammonium salt, M_N-2-HACC_ = 328.5; and M_CS_ is the molecular weight of the chitosan monomer, M_CS_ = 161.

### 4.2. Water Solubility and Antibacterial Activity of Different Degrees of Substitution of N-2-HACC

The water solubility of N-2-HACC was evaluated by light transmittance; 20 mg of N-2-HACC (50%, 60%, 70%, and 80%) was dissolved in 5 mL of ddH_2_O. Each sample was used for measuring light transmittance, and ddH_2_O was used for the control group.

To obtain the best antibacterial effects of different degrees of substitution of N-2-HACC against *S. mutans*, 0.5 mg/mL N-2-HACC with 50%, 60%, 70%, and 80% degrees of substitution was incubated with 1 × 10^6^ CFU/mL *S. mutans* at 37 °C for 12 h. The positive group was a culture medium and *S. mutans*, while the negative group was only a culture medium. The OD_600_ of the bacterial suspension was measured to calculate the antibacterial rate (AR) (2).

(2) $$\mathrm{AR}=\left({\mathrm{OD}}_{\mathrm{positive}\;\mathrm{group}}-{\mathrm{OD}}_{\mathrm{tested}\;\mathrm{group}}\right)÷\left({\mathrm{OD}}_{\mathrm{positive}\;\mathrm{group}}-{\mathrm{OD}}_{\mathrm{negative}\;\mathrm{group}}\right)\times 100\%$$

### 4.3. Determination of Minimum Inhibitory Concentration (MIC)

MIC was analyzed using the microdilution method to determine the antibacterial activity of N-2-HACC against *S. mutans* [29]. N-2-HACC was dissolved in Brain Heart Infusion (BHI, Guangzhou Huankai Biotechnology Co., Ltd., Guangzhou, China), and 0.1 mL of each N-2-HACC sample was added to a 96-well plate, along with 0.1 mL of *S. mutans* (1 × 10^6^ CFU/mL). Two-fold serially diluted N-2-HACC was used to create different types of solutions with final concentrations of 10 mg/mL, 5 mg/mL, 2.5 mg/mL, 1.25 mg/mL, 0.625 mg/mL, 0.3125 mg/mL, 0.156 mg/mL, and 0.078 mg/mL. The mixture was incubated at 37 °C for 24 h. The MIC of N-2-HACC was determined by measuring the absorbance value of the bacterial suspension at 600 nm.

### 4.4. N-2-HACC Time-Kill Curves

N-2-HACC was dissolved in water to obtain a 1× MIC solution. Here, 1 × 10^6^ CFU/mL of *S. mutans* was treated with 1× MIC N-2-HACC in the treated group and 0.9% NaCl in the control group in a 96-well plate. Then the mixture was incubated at 37 °C for 48 h. Absorbance at 600 nm was measured at time intervals of 0, 2, 4, 8, 12, 24, and 48 h in each well using a microplate reader [12]. All the experiments were independently repeated at least three times.

### 4.5. Scanning Electron Microscopy (SEM) Observation

*S. mutans* was incubated with N-2-HACC concentrations of 1× MIC or 0.9% NaCl at 37 °C for 24 h. The mixture was centrifuged at 4000 rpm for 10 min, followed by washing with PBS three times, fixing with 2.5% glutaric dialdehyde, dehydrating with 30%, 50%, 70%, 80%, 90%, or 100% absolute ethyl alcohol, and drying overnight. Finally, the samples were observed under a scanning electron microscope.

### 4.6. Detection of Permeability of Cell Wall

Alkaline phosphatase (AKP) was detected to evaluate the permeability of the cell wall of *S. mutans* after incubation with 1× MIC N-2-HACC or 0.9% NaCl at 37 °C for 0, 2, 4, 6, and 8 h. The procedure for each group was performed in triplicate. After incubation, the mixed solution was centrifuged at 5000 rpm for 10 min. The supernatant was subjected to AKP detection according to the instructions of the Alkaline Phosphatase Assay Kit (Beyotime, Shanghai, China) [31].

### 4.7. Effect of N-2-HACC on the Cell Membrane of S. mutans

#### 4.7.1. Propidium Iodide (PI) Staining

*S. mutans* (1 × 10^6^ CFU/mL) was incubated with a 1× MIC concentration of N-2-HACC at 37 °C for 2 h; 0.9% NaCl was used for the control group. The procedure for all samples was repeated three times, and the samples were centrifuged at 6000 rpm for 10 min; the sediment was washed with PBS three times, then stained at 37 °C for 30 min using the Annexin V-FITC/PI Apoptosis Detection Kit (Vazyme Biotech Co., Ltd., Nanjing, China) and observed under a fluorescent microscope.

#### 4.7.2. Conductivity Measurement

*S. mutans* (1 × 10^6^ CFU/mL) was incubated with 1× MIC concentrations of N-2-HACC or 0.9% NaCl at 37 °C for 6 h. The procedure was repeated at least three times for all groups. Finally, the conductivity of the supernatant of the bacteria was measured at time intervals of 0, 1, 2, 3, 4, 5, and 6 h in a conductivity meter.

#### 4.7.3. Detection of Nucleic Acid in BHI of *S. mutans*

*S. mutans* (1 × 10^6^ CFU/mL) was incubated with 1× MIC N-2-HACC in the treated group and 0.9% NaCl in the control group at 37 °C for 3 h. OD_260_ was measured at time intervals of 0, 0.5, 1, 1.5, 2, 2.5, and 3 h [34,36]. Experiments were performed in triplicate, and each was repeated at least three times.

#### 4.7.4. Detection of Metabolic Activity of *S. mutans*

*S. mutans* (A_600_ = 0.5) was incubated with 1× MIC concentrations of N-2-HACC and 0.9% NaCl at 37 °C for 3 h and 12 h, respectively. Then, 150 μL of iodonitrotetrazolium chloride (0.67 mg/mL, Solarbio Science & Technology Co., Ltd., Beijing, China) was mixed with 1350 μL of the above bacterial suspension and incubated at 37 °C for 30 min, and the absorbance at OD_630_ was measured [38]. The procedure for each group was performed in triplicate.

### 4.8. Effect of N-2-HACC on the Respiratory Metabolism of S. mutans

*S. mutans* (1 × 10^6^ CFU/mL) was incubated with 1× MIC N-2-HACC at 37 °C for 12 h. 0.9% NaCl with *S. mutans* was used as the control group. Na^+^-k^+^-ATP enzyme (Shanghai Tongwei Biotechnology Co., Ltd., Shanghai, China), pyruvate kinase (PK) (Nanjing Jiancheng Bioengineering Institute, Nanjing, China), succinate dehydrogenase (SDH) (Nanjing Jiancheng Bioengineering Institute, Nanjing, China), and malate dehydrogenase (MDH) (Shanghai Tongwei Biotechnology Co., Ltd., Shanghai, China) were detected by the corresponding kits to evaluate the effects of N-HACC on the respiratory metabolism of *S. mutans*. The procedures for the treated group and the control group were performed in triplicate.

### 4.9. Effect of N-2-HACC on Contents of DNA and Soluble Protein of S. mutans

The DNA of *S. mutans* was extracted by TIANamp Genomic DNA Kit (Tiangen, Co., Ltd., Beijing, China) according to the manufacturer’s instructions. Then, 1× MIC concentrations of N-2-HACC were mixed with the DNA of *S. mutans* and incubated at 37 °C for 30 min. Here, a fluorospectrophotometer was used to detect the fluorescence spectrum of the EB-DNA-N-2-HACC mixture at an excitation wavelength of 480 nm and an emission wavelength of 300–700 nm.

Next, 1× MIC concentrations of N-2-HACC and 0.9% NaCl were mixed with *S. mutans* and incubated at 37 °C for 12 h. The bacterial suspension was treated with an ultrasonic cell pulverizer, and the BCA Protein Assay Kit was used to determine the concentration of protein that leaked from *S. mutans* [36].

### 4.10. Detection of S. mutans Biofilm Formation

For the biofilm inhibition rate, 1× MIC concentrations of N-2-HACC were mixed with *S. mutans* and incubated at 37 °C for 12 h, respectively. For mature biofilm inhibition rate detection, *S. mutans* was cultured at 37 °C for 24 h first, and then the bacterial suspension was incubated with 1× MIC concentrations of N-2-HACC at 37 °C for 24 h. Here, 0.9% NaCl with *S. mutans* was used as the control group. All the samples of supernatant were discarded, and then the sediment was fixed in 4% paraformaldehyde for 4 h and strained with crystal violet for 30 min. Finally, samples were washed with PBS three times, dried, and dissolved in 30% acetic acid. OD_600_ was measured to calculate the biofilm inhibition rate, as in a previous study. Biofilm formation was observed under a fluorescent microscope [11,39]. The experiments were repeated at least three times.

### 4.11. Effect of N-HACC on the Virulence Genes of S. mutans

To evaluate the transcription levels of *vick* and *ftf* mRNA, 1× MIC concentrations of N-2-HACC were mixed with *S. mutans* and incubated at 37 °C for 12 h. The total RNA of the control (0.9% NaCl) and treated groups was extracted using the TRNzol Universal total RNA kit (Tiangen, Co., Ltd., Beijing, China). Specificity primers were designed according to the corresponding sequences in GenBank (Table 1). The 16S gene was used as the housekeeping gene. qRT-PCR was performed in triplicate using SYBR Premix Ex TaqTM II (Perfect Real Time) (Takara, Japan).

### 4.12. Statistical Analysis

All the data in the present study are presented as the mean ± the standard deviation (SD). All the samples were assessed in triplicate. One-way analysis of variance (ANOVA) and multiple comparison tests (Tukey) were used to analyze and compare the data. A difference of *p* < 0.05 was considered statistically significant.

## 5. Conclusions

In summary, the present study provides evidence that N-2-HACC has a strong antimicrobial activity against *S. mutans* via cell membrane integrity damage, increasing the permeability of cell walls, interfering with bacterial protein and nucleic acid synthesis, perturbing glycolysis and the TCA cycle, and inhibiting biofilm formation. Therefore, N-2-HACC has remarkable proficiency as a natural antibacterial agent, and it also has the potential to be used as an effective synthetically modified natural antimicrobial material.

## Figures and Tables

**Table 1:** Primers used in the present study.

Genes	Primers Sequence (5′-3′)	
*ftf*	*ftf*-F	AAATATGAAGGCGGCTACAACG
	*ftf*-R	CTTCACCAGTCTTAGCATCCTGAA
*vick*	*vick*-F	ATGGCGGTAAGGTGACTG
	*vick*-R	GACCCTTCGCCTTCCTCACTA
16S	16S-F	CCTACGGGAGGCAGCAGTAG
	16S-R	CAACAGAGCTTTACGATCCGAAA

## Data Availability

Data are contained within the article.
